# PhyLab – a virtual reality laboratory for experiments in physics: a pilot study on intervention effectiveness and gender differences

**DOI:** 10.3389/fpsyg.2024.1284597

**Published:** 2024-02-19

**Authors:** Selma Korlat, Marlene Kollmayer, Christian Haider, Helmut Hlavacs, Daniel Martinek, Patrick Pazour, Christiane Spiel

**Affiliations:** ^1^Department for Psychology of Development and Education, Faculty of Psychology, University of Vienna, Vienna, Austria; ^2^Entertainment Computing, Faculty of Computer Science, University of Vienna, Vienna, Austria

**Keywords:** game-based learning, virtual reality, physics laboratory, intervention, gender differences

## Abstract

**Introduction:**

New technologies have great potential to facilitate students’ understanding and appreciation of one of the most abstract and challenging school subjects – physics. This study aimed to examine the effects of a game-based virtual reality teaching method on secondary school students’ self-beliefs, interest, and performance in physics through a quasi-experimental design using pre- and post-test data. The evaluation is based on the systemic actiotope model that explains a person’s goal-oriented actions by an interplay of their environment, action repertoire (i.e., students’ performance and interest in physics), and subjective action space (i.e., students’ self-efficacy, self-concept, and implicit theories regarding physics).

**Method:**

A game-based virtual reality App to be used with Google cardboards was developed containing 10 teaching units from the secondary school physics class curriculum. Participants in the control group were taught using traditional teaching methods, while students in the experimental group went through the VR with the teacher and conducted the prepared VR experiments in addition to the traditionally presented content. Three tests measured students’ physics performance during the semester. In addition, students answered questionnaires assessing their interest, self-efficacy, self-concept, and entity implicit theories regarding physics before and after the intervention, resulting in a Pretest-Posttest Control Group Design.

**Results:**

There were no significant differences between the control and experimental group in test scores on the first and second tests but compared to the control group, the experimental group achieved higher scores on the third test. In addition, the results indicate differential effects of the game-based virtual reality teaching method on students’ interest and self-efficacy regarding physics to the advantage of students identifying as male, but no effects on students’ self-concept, and entity implicit theories regarding physics.

**Discussion:**

The results of our pilot study suggest that incorporating innovative didactic methods into secondary school physics classes could potentially contribute to higher performance in and motivation for physics during this crucial period of adolescence when students develop educational and career aspirations. However, game-based virtual reality teaching methods seem to favor students identifying as male, which should be considered in their development and presentation. Other practical implications for practitioners and researchers are discussed.

## Introduction

In the last few decades, there is a consistent growth of engineering and technical industry. Consequently, the importance of skills, as well as the earning potential of careers in physics and related STEM (science, technology, engineering, and mathematics) fields is increasing (Diekman et al., 2017). Nevertheless, data consistently shows decreasing numbers of students who pursue scientific degrees and are interested in STEM fields (Vu and Feinstein, 2017). The declining course enrollment in physical science subjects is due to the high perceived difficulty of these subjects even among high achieving students (Osborne et al., 2003; Lyons, 2006). Indeed, studies suggest that students perceive physics as one of the most difficult and most disliked of all school subjects (Osborne et al., 2003; Kessels, 2005). Accordingly, research has found that interest in physics continuously declines from ages 11–16 (Bennett and Hogarth, 2009; Vahedi and Yari, 2014). Against this backdrop, scholars have called for interventions to motivate students for physics’ learning (Graves et al., 2017), especially in the period of the transition to secondary school, (Wang et al., 2017) when career aspirations are being developed and educational trajectories are being chosen (Eccles, 2009). This is particularly important among girls, as women are significantly underrepresented in physics and STEM-related fields compared to men (Eccles, 2009), which is already visible in their lower occupational aspirations in these fields during adolescence (e.g., OECD, 2012; Wegemer and Eccles, 2019). Intervention programs promoting physics engagement should promote performance and motivational factors in a comprehensive way to improve students’ education and career prospects regardless of gender and also be innovative and entertaining for students. Thereby new technologies have great potential to facilitate students’ understanding and appreciation of physics (e.g., Wieman and Perkins, 2005; Chandra and Watters, 2012). The goal of this study is to examine the effects of a game-based teaching method using virtual reality on secondary students’ performance, interest and self-beliefs regarding physics.

### Physics in secondary school education

Learning to solve physics problems is an important goal of secondary education (Pol et al., 2008). Students, however, perceive physics as a demanding, theoretical, abstract, and labor-intensive subject (Angell et al., 2004). Many of them struggle to solve problems in which physics knowledge must be applied (Pol et al., 2008). Kessels (2005) used the implicit association test and showed that students associate physics with difficulty more than ease and with unpleasantness more than pleasure. This seems to be particularly pronounced among girls who often report lower beliefs in their abilities and lower fitness in physics than boys (e.g., Nissen and Shemwell, 2016; Henderson et al., 2020). A binary representation of gender, as represented in these studies, is increasingly criticized, and more and more young people in Western Europe are identifying as non-binary (e.g., Paechter et al., 2021; Bower-Brown et al., 2023). Current approaches to analyzing gender explicitly incorporate “ideas of the fluidly embodied, socially constructed, and self-constructed aspects of social identity, along with the dynamic interaction and integration of these aspects of identity within the narratives of lived experiences” (Nagoshi and Brzuzy, 2010, p. 432). Nevertheless, the existing research on attitudes toward and motivation for physics focuses on differences between students identifying as male or female and traditional gender stereotypes in this domain.

Given its complexity and abstractness, it might be difficult for students to grasp and capture the link between the theoretical scientific background and physical representation of physics concepts. Accordingly, Dounas-Frazer and Lewandowski (2018) suggested explicitly supporting and visualizing such links during experimenting processes (Thees et al., 2020). Moreover, learning scientific concepts in physics from different modes in which the content is presented and explained has been shown to be highly beneficial for students’ understanding (Etkina et al., 2006; Treagust et al., 2017). However, the secondary school curriculum in Austria, unlike university laboratory courses, includes little experimental work and only a few opportunities for students to experience physics phenomena. Thus, traditional teaching methods at the secondary school level might be unsuitable for a deep understanding of physics concepts, especially in a rapidly evolving, technology-saturated world (Figueroa-Flores, 2016).

### Game-based learning

With the advancement in technology in the past decades, new forms of teaching have emerged (Hussein and Natterdal, 2022), introducing gaming in primary and secondary school education (e.g., Rachels and Rockinson-Szapkiw, 2018; Zainuddin, 2018; Ioannou, 2019), as well as higher education (e.g., Huang and Hew, 2018) to promote desired behavior and learning outcomes (Zainuddin, 2018). The term game-based learning (GBL) refers to the utilization of games in education. “Serious games” aim to achieve defined learning outcomes by involving playful problem-solving challenges which provide learners, who are also players, with a sense of achievement (Qian and Clark, 2016). These didactic practices have been found to be accompanied by positive educational attainment, such as knowledge acquisition and content understanding (Connolly et al., 2012; Vlachopoulos and Makri, 2017), as well as academic performance improvement (for a systematic review see Krath et al., 2021). For example, Chung and Chang (2014) investigated acquisition of first aid knowledge and language skills and found that the learning achievements of both genders in the experimental group who learned with GBL method were significantly higher than those of the control group that was taught by the traditional teaching method. Similarly, Chung and Chang (2014) showed that students who learned natural science content using the GBL environment significantly outperformed their peers who learned with the conventional e-learning approach. A systematic review (Boyle et al., 2016) comprising 129 papers ranging from 2004 to 2009 and 143 papers from 2009 to 2014 showed that using serious games not only promoted students’ knowledge acquisition and performance but also led to positive behavioral changes in terms of cognitive, motivational, emotional, and social benefits. Previous studies applying GBL in STEM subjects confirmed its positive effects (e.g., Vu and Feinstein, 2017; Hussein et al., 2019). For instance, Sung and Hwang (2013) reported that students who learned with serious games had significantly higher learning performance and attitude toward science compared to students in the control group who played the standard version of the game. A recent study that focused on GBL in physics (Kao et al., 2017) showed that students who learned with GBL scored significantly higher on their concept maps (knowledge acquisition measure) than those who did not use the game.

### Virtual reality learning

Recently, game-based virtual reality (VR) learning environments, where learning content is incorporated into gameplay accessed through virtual reality, have garnered much attention among researchers due to their combination of usability and likability (Virvou and Katsionis, 2008). Virtual reality simulates or replicates aspects of the physical world (Makransky and Lilleholt, 2018), and can be presented through both non-immersive monitor interfaces and immersive head-mount devices (HMDs). This technology engenders realistic and engrossing experiences, expanding the scope of scenarios compared to traditional real-life teaching (Howard and Gutworth, 2020). The application of VR in education can help to provide more active, constructivist learning, increase the frequency of authentic learning experiences, and provides an arena for visualizing abstract concepts concretely (Hu Au and Lee, 2017), thereby promoting students’ engagement and motivation (Araiza-Alba et al., 2021; Shi et al., 2022). As such, game-based virtual reality learning environments seem to be a well-suited medium for facilitating students’ comprehension of physics concepts. Especially with the increasing accessibility of Google Cardboard and its applicability and user-friendliness with any smartphone, VR became an obtainable and convenient instrument for newer generations’ learning process in and outside of the classroom (Hussein and Natterdal, 2022). Thus, the utilization of such tools provides an opportunity of conducting experimental lab work in secondary school classes. Moreover, it could contribute to a decrease in gender disparities in this context, as significant gender differences in participation in lab work have been shown, with men mainly taking over the equipment and management of the apparatus in the onsite setting (Holmes et al., 2014).

Studies integrating VR with GBL found significant improvement in students’ learning motivation, positive learning experiences (Christopoulos et al., 2023) and learning outcomes, such as learners’ ability in recall processes, as well as enhancement of their cognitive thinking and problem-solving abilities (Shi et al., 2019). Previous studies have shown clear advantages of VR across different STEM fields in terms of students’ positive attitudes, engagement, and performance (for a review see Pellas et al., 2020). New studies that focused specifically on physics (Bogusevschi et al., 2020) showed a significantly greater knowledge gain in students using a VR application compared to students who learned without VR. Pirker et al. (2022) reported increased motivation in students who used VR in physics education, as well as higher interest and positive emotions related to VR compared to traditional teaching methods.

### Theoretical framework

The attempts to increase students’ proactivity in physics should focus not only on enhancing students’ current performance, but also promote the factors that ensure long-term engagement in the field. Against this backdrop, the actiotope model that explains human actions based on system theory (Ziegler et al., 2006, 2011) serves as a helpful framework for comprehensively evaluating intervention effectiveness (e.g., Kollmayer et al., 2019). According to the model, an individual’s actiotope consists of four interacting components: environment, goals, action repertoire, and subjective action space. The environment refers to the material and symbolic framework for an individual’s goal-oriented actions. Goals can be defined as an individual’s ambitions or desired results in a certain area of life. The action repertoire characterizes all forms of goal-oriented behavior which an individual is theoretically capable of performing. The subjective action space contains all behavior a person perceives as feasible for themselves. According to the actiotope model, the process of transforming the current situation into a desired future state is regulated by the individual’s action repertoire, subjective action space and the behavior options given in a specific environment (Kollmayer et al., 2020). Thus, students’ learning environment in physics should promote their action repertoire and subjective action space in order to affect their educational and career goals in this field.

**Figure 1:**
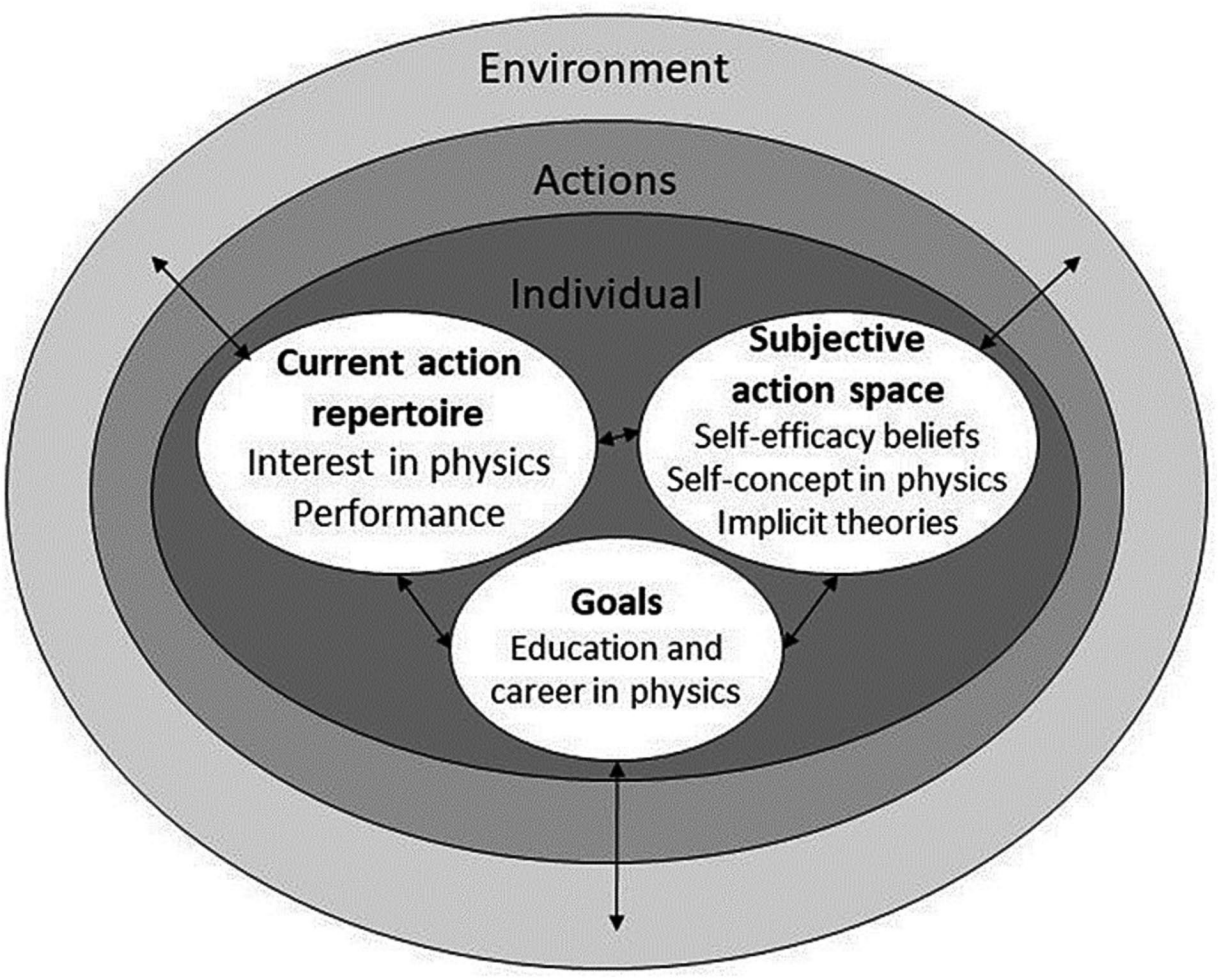
The actiotope model.

In our study, students’ action repertoire is represented by their academic performance and interest in physics, while their subjective action space is represented by their self-efficacy, academic self-concept, and implicit theories regarding physics (see Figure 1). Previous studies indicated self-efficacy (one’s judgment of how well or poorly one will cope with a situation or task; Bandura, 2001), academic self-concept (a description of one’s own perceived self in a given achievement situation which may include an evaluative judgment of self-worth; Guay et al., 2004) and entity implicit theories (beliefs that abilities in a specific field cannot be changed; Dweck et al., 1995) as self-beliefs that that strongly influence students’ persistence and later attainment in STEM field (e.g., Bong and Skaalvik, 2003; Fencl and Scheel, 2005; Cury et al., 2006; Shively and Ryan, 2013; Dai and Cromley, 2014; Sax et al., 2015). In addition to that, interest, as a content-specific motivational characteristic composed of intrinsic feeling-related and value-related valences, has been confirmed as an important predictor of student achievement, further engagement in advanced subject courses, and future career aspirations in STEM fields (e.g., Kang and Keinonen, 2018). A game-based VR learning environment incorporated in the traditional school environment as a new setting could promote students’ action repertoire and subjective action space, thus facilitating career and education goals in physics.

### Present study

This quasi-experimental study aimed to integrate game-based VR learning in secondary school physics curriculum to improve students’ actual action repertoire and subjective action space in this subject. First, we explored associations between the different facets of students’ actiotope to allow for deeper insights into students’ physics motivation (Ziegler et al., 2006, 2011). A recent systematic review (Krath et al., 2021) showed that different theories of motivation and learning have been used to explain the positive effects of gamification, and that they share important conceptual connections: Game-based learning can illustrate learning goals and their relevance and support users in setting individual goals. Moreover, gamification can promote learning by providing specific learning paths and immediate feedback, reinforcing good performance, and helping to better divide and organize learning content. These principles have a positive impact on performance and a broad range of motivational variables. Therefore, we formulated the following hypotheses considering the reported advantages of VR and game-based learning for students’ learning performance and motivation (Zainuddin, 2018; Krath et al., 2021): The experimental group will perform significantly better on physics tests than the control group (H1). The experimental group will report a stronger increase in interest (H2), self-efficacy (H3), and academic self-concept in physics (H4) than the control group–over time. The experimental group will report a significantly lower decrease in entity implicit theories than the control group over time (H5). Finally, we explore, whether using the game-based VR App has differential effects on students of different gender.

## Method

### Data collection

Data was collected in the summer semester of 2021, with the pre-test (questionnaire assessment before the intervention) taking place at the beginning of the semester in March and the post-test (questionnaire assessment after the intervention) taking place at the end of the semester in June. The study sample in the pre-test consisted of 70 fourth graders (45.7% identifying as female and 54.3% identifying as male, *M*_age_ = 14.09, *SD*_age_ = 0.78; age-range 13–15) from a compulsory secondary school in Vienna predominantly attended by children from middle SES families. The post-test sample comprised 55 fourth graders from the same school (45.5% identified as female, 52.7% as male, and 1.8% as non-binary, *M*_age_ = 14.31, *SD*_age_ = 0.74; age-range 13–16). Only students who gave active consent were included in the data set. Anonymity and confidentiality of their data were guaranteed. Due to dropouts, the final sample of students who answered the questionnaire at both measurement times consisted of 43 students (30 from the control group and 23 from the experimental group; 23 identified as male and 20 identified as female).

In addition, we use the results from three tests with 77 students (57 from the control group and 20 from the experimental group; 31 identifying as male, 30 identifying as female, 16 missing) having completed the first test, 68 students (50 from the control group and 18 from the experimental group; 32 identifying as male, 27 identifying as female, 9 missing) having completed the second test, and 61 students (46 from the control group and 15 from the experimental group; 33 identifying as male, 28 identifying as female) having completed the third test.

### VR lab design

In 2020, a game-based virtual reality App was developed containing 10 teaching units from the secondary school physics class curriculum: electricity, electrical voltage, electrical currency, electromagnetic spectrum, forces, climate, energy, temperature, magnetic coil and radioactivity. Table 1 presents learning material and information the App contained within each teaching unit. In addition to the interactive experiment, each unit contained a quiz with five questions on information presented in the individual unit. Students were able to accumulate numeric points in the learning process, as well as level up in the game and follow their own progress in a progress bar (see examples of the VR settings in Supplementary Figures S1–S9).

**Table 1:** Topics and information covered in VR physics teaching units.

Topic	Thematic unit	VR setting	Experiment topic and design	Interactive information included
Electricity	Noble and base metals	Classroom	Students try out different types of metals in the process of building batteries and their functioning	Volta column, Alessandro Volta, battery recycling and lithium mining problematics
Electrical voltage	Noble and base metals	Electrical factory with surrounding thunderstorms	Students can experiment with simple, parallel, serial and series circuits	Georg Simon Ohm, electrical plugs, wind turbines and Ohm’s law
Electrical currency	Noble and base metals	On top of a huge building in the city	An atomic water jet experiment that explains excess electrons; students add or subtract free electrons to a wand to deflect the jet of water	Benjamin Franklin and electric charge
Electromagnetic spectrum	World of light	Mountain forest area	Individual areas of the spectrum can be made “visible” in order to see which everyday objects emit which radiation	Radiation exposure, prisms, space telescopes and Joseph Fraunhofer
Forces	World of light	Different planets with different gravitational forces	Weight of 1 kg on a spring in a forest on Earth, a crater on the Moon, a dessert on Mars and on the surface of Jupiter	Sir Isaac Newton and gravitation
Climate	World of light	Spacecraft in the Earth’s stratosphere with simulation of global warming	Students can influence various factors such as forest planting adding or removing coal and wind power plants to observe how the simulated world and the CO_2_ household changes	Greta Thunberg, carbon dioxide and the ozone layer
Energy	World of light	Small amusement park	A roller coaster experiment shows the connection between kinetic and potential energy; students can choose different vehicles to experience different relations	Pendelum clocks, Hermann Helmholtz and forms of energy
Temperature	World of light	Ice cave	The connection between temperature and the state of aggregation is explained using the example of water; the temperature of a pool of water can be set from absolute zero to several hundred degrees Celsius and the movement of the molecules is made visible	William Thomson, glaciers and heat transfer
Magnetic coil	Noble and base metals	Factory building	The experiment shows how the electric and magnetic fields interact in coils; different coils can be combined and the resulting field becomes visible	Emil Lenz, Farady cage and Michael Faraday
Radioactivity	Radioactivity	Radioactive power plant	Different materials can be tested in the experiment for their permeability against different types of radiation	Nuclear fission, Lise Meitner, atomic energy and the special theory of relativity

The units of the app were developed in individual sprints with very close thematic cooperation with the physics teacher. The units in the App were unlocked at the same time as the thematic was planned in the curriculum. When the App is opened, the first interface is a hub area with portals to each unlocked unit and an introduction is made by an App-integrated robot character. Throughout the app the students are accompanied by that flying robot which acts as the storyteller, quiz master and helper with introductions to the app spaces and each interactive experiment. All the information is fully voice recorded and communicated through the companion robot with a robotic voice effect.

The VR application was developed using the Unity 3D Engine with the LTS Version ‘2018.4’ and the Google VR SDK for Unity. For the building of the level geometry the Unity tools Polybrush and ProBuilder were used. While the more general models like trees and grass were imported from free assets on the Unity Asset Store, all the specific models and animations for the interactive experiments and the robot companion were created specifically for this project by hand with blender. All of the audio recordings were created with the free open-source software Audacity. For the data collection the VR application sent its information as JSON encoded data via HTTPS calls to a simple REST API running on a shared web server by the university.

### Intervention implementation

The App was used with Google cardboards, which were provided for all students in the experimental group. As the App was developed for the Android operating system, participants with iOS mobile phones and a few participants who did not possess a mobile phone were assigned to the control group, whereas students with mobile phones with Android operating system were randomly assigned to both groups resulting in an approximately equal percentage of students in both groups. However, due to the pandemic context, not all students from the experiment group used the App, nor all of the students participated in both assessments, resulting in *n* = 19 participants in the experimental group and *n* = 51 in the control group in pre-test, and *n* = 15 participants in the experimental and *n* = 40 in the control group in post-test. The group assignment was done by the school principal and physics teacher. They assigned codes to the students, which they used in questionnaires, on tests, and in the App. The authors did not have access to the codes until the study was finished.

During the summer semester of 2021, participants in the control group were taught using traditional teaching methods whereby the teacher presented the content of the week’s teaching unit verbally in the classroom using the textbook and related teaching materials without any GBL or VR elements. The experimental group participated in the equally designed classes as the control group, but in addition to the traditionally presented content, the teaching unit material was demonstrated through the App in the classroom, whereby students went through the VR with the teacher and conducted the prepared VR experiments. The students from the experimental group also had the opportunity to use the App after the class to access VR laboratory while doing the homework, which was recommended but not mandatory nor controlled for.

Although data collection took place during a semester in which there were school closures due to the COVID-19 pandemic, during the study implementation teaching was conducted face-to-face in smaller groups. Hence, all participants under the study had equal presence-based teaching conditions. In both pre-and post-test, participants answered an online questionnaire containing the same items via Unipark (Questback GmbH, 2016). Additionally, at the end of the semester, after three broader thematic units (noble and base metals, world of light and radioactivity), participants took short performance tests evaluating the knowledge related to each thematic unit (see T1, T2 and T3 in the supplementary material for task examples on the tests, and answered few questions on their experience with the unit and with the App (the latter was asked only in experimental group). To account for possible implementation effects in the effectiveness of the intervention, user behavior data was tracked in the App during the whole semester (Schultes et al., 2015).

### Measures

Since schools have little time for extracurricular activities due to the pressure to fulfill the curriculum, the survey instruments were designed to be as economical as possible in order to keep the data collection effort for students and teachers as low as possible and, at the same time, be able to make reliable statements. Students’ physics performance was measured with three short tests related to thematic units covered in the App at the end of the semester. All other actiotope variables were assessed at the beginning and the end of the semester using items from standardized instruments selected based on test-theoretical considerations. All items in the questionnaire were rated on a 5-point Likert-type scale, ranging from 1 (strongly agree) to 5 (strongly disagree). In order to simplify the interpretation of results, all analyses were conducted with recoded items so that higher values reflected higher agreement with the statements.

### Actiotope variables

#### Students’ action repertoire

Students’ action repertoire was operationalized by their performance and interest in physics.

*Physics performance* was measured with three short tests designed by the teacher with regard to three thematic teaching units covered with the App (“noble and base metals”; “world of light”; and “radioactivity”). Each test consisted of two tasks directly relating to the learning contents of the respective teaching unit (see Supplementary Figures S10–S12). The tests were corrected and evaluated by the teacher.

*Interest in physics* was measured with four items from Krapp (2002), sample item: “Sometimes when I do a task in physics, I forget everything around me.”; *α* = 0.780 in pre-test and *α* = 0.805 in post-test.

#### Students’ subjective action space

Students’ subjective action space was operationalized by their self-efficacy, self-concept and implicit theories regarding physics.

To assess students’ *self-efficacy* in physics, two standardized items from Kunter et al. (2000) and one from Jerusalem and Satow (1999) were used to capture beliefs in their ability to succeed in or accomplish a task (sample item: “I am convinced that I can do well on class assignments and tests in physics; *α* = 0.823 in pre-test and *α* = 0.858 in post-test).

The academic self-concept scale (Dickhäuser et al., 2002) was used to measure *academic self-concept* based on no reference norms (four items). The items were adapted to Physics (sample item: “A am for physics… 1-very talented, 5-not talented; *α* = 0.924 in pre-test and *α* = 0.926 in post-test).

To test students’ implicit theories regarding whether or not abilities in physics are malleable, four items from Wagner et al. (2010) were used (sample item: “I cannot change the fact that there are things in physics that I just cannot do; *α* = 0.893 in pre-test and *α* = 0.918 in post-test).

### Data analysis

IBM SPSS 29 was used to conduct the data analysis. The dataset underwent a thorough review for inconsistencies to ensure usability. Only complete cases were considered for analyses. To test our hypotheses, we performed *t*-tests to analyze differences between the control group and the experimental group in physics performance and mixed ANOVAs to analyze changes in all other actiotope variables after testing for assumptions.

## Results

### Descriptive analysis

#### Participant responsiveness

The number of seconds the Apps was open in the foreground and the number of interactions made within the App were two main indicators of participants’ responsiveness tracked in the App. In total, data from 22 participants was tracked in the App. On average, App was open 12.43 min in the foreground (*SD* = 10.41), ranging from 1.5 to 33 min. On average, participants made 23.64 interactions in the App (*SD* = 29.62), ranging from 1 to 116 interactions.

#### Associations between different actiotope components

Table 2 provides bivariate correlations among all the actiotope variables pre-and post-test. Interest, self-efficacy and self-concept were highly interrelated at both measurement points. Entity implicit theories showed negative correlations with both self-efficacy and self-concept at the pretest, but only with self-concept at the posttest, while no significant correlations with interest were found at any measurement point.

**Table 2:** Bivariate correlations between actiotope variables at the pre- and the posttest.

Variables		1	2	3	4
1. Interest	Pearson-Correlation	–	0.449^**^	0.515^**^	0.110
	Significance (two-tailed)		0.001	<0.001	0.424
	N		55	55	55
2. Self-efficacy	Pearson-Korrelation	0.530^**^	–	0.763^**^	−0.235
	Significance (two-tailed)	<0.001		<0.001	0.084
	N	70		55	55
3. Self-concept	Pearson-Korrelation	0.66^**^	0.768^**^	–	−0.333^*^
	Significance (two-tailed)	<0.001	<0.001		0.013
	N	70	70		55
4. Implicit entity theory	Pearson-Korrelation	0.047	−0.389^**^	−0.477^**^	–
	Significance (two-tailed)	0.699	0.001	<0.001	
	N	70	70	70	

Correlations between actiotope variables at the pretest are below the diagonal. Correlations between actiotope variables at the posttest are above the diagonal.

^**^0.01 (two-tailed). ^*^0.05 (two-tailed).

### Differences in physics performance

Dependent *t*-tests were performed to analyze whether there was a performance difference in knowledge between the control and the experimental group in the three administered physics tests. There were no significant differences between the control and experimental group in test scores on the first (“noble and base metals” thematic unit) and second (“world of light” thematic unit) tests. Results showed that, compared to participants in the control group, participants in the experimental group achieved higher scores on the third test (“radioactivity” thematic unit). Results showed no significant differences between boys and girls in all three tests (see Table 3).

**Table 3:** Mean, standard deviation and *t*-test statistics for experimental and control group and for boys and girls on physics tests.

	E		C					
	*M^a^* (*SD*)	*n*	*M^a^* (*SD*)	*n*	*t*	*d*	*p*	CI 95%
Test1	65.75 (19.35)	20	65.44 (21.98)	57	−0.056	0.01	0.955	(−11.36|10.74)
Test2	46.41 (16.63)	18	46.62 (13.98)	50	0.051	0.01	0.960	(−7.87|8.28)
Test3	97.22 (4.04)	18	91.09 (16.10)	43	−2.330	0.52	0.024	(−11.42|−0.85)
	Girls		Boys					
	*M^a^* (*SD*)	*n*	*M^a^* (*SD*)	*n*	*t*	*d*	*p*	CI 95%
Test1	62.00 (20.87)	30	66.13 (20.52)	31	0.779	0.19	0.439	(−6.48|14.73)
Test2	47.07 (12.62)	27	46.15 (16.74)	32	−0.233	0.06	0.817	(−8.76|6.94)
Test3	96.47 (5.86)	26	90.48 (16.62)	28	−0.375	0.48	0.082	(−12.80|0.80)

E, experimental group; C, control group; d, Cohen’s d.

^a^Total test score = 100%.

### Development of interest, self-efficacy, self-concept, and implicit theories regarding physics

To test our hypotheses a sequence of mixed ANOVAs was conducted with the measurement points (pretest and posttest) as with-in subject factor and the groups (experimental group and control group) and gender (female and male) as between subject factors, and with students’ interest, self-efficacy, self-concept, and implicit entity theories as dependent variables. Interaction effects and main effects are reported. To identify any outliers, boxplot diagrams were utilized. Two outliers were determined in the lower spectrum of self-efficacy and self-concept by a single individual in the control group, while two outliers were detected in the high-end spectrum of self-concept also in the control group. Despite their presence, these values are deemed important as they fall within the range of the scale, and therefore were not excluded from data analysis. Furthermore, Levene tests were performed for each actiotope variable to test for equality of variances and Box-tests for equality of the covariance matrixes. Descriptive statistics for all dependent variables are depicted in Table 4.

**Table 4:** Descriptive statistics of actiotope variables in pre- and posttest by group and gender.

			Pretest			Posttest		
Actiotope variable	Group	Gender	*M*	*SD*	*n*	*M*	*SD*	*n*
Interest	Control group	Male	2.95	0.73	16	2.38	0.99	16
		Female	2.49	0.59	14	2.71	0.81	14
		Total	2.74	0.70	30	2.53	0.91	30
	Experimental group	Male	3.11	0.78	7	3.05	0.47	7
		Female	3.13	0.96	6	2.79	1.05	6
		Total	3.12	0.83	13	2.93	0.77	13
	Total	Male	3.00	0.73	23	2.58	0.91	23
		Female	2.68	0.75	20	2.74	0.86	20
		Total	2.85	0.75	43	2.65	0.88	43
Self-efficacy	Control group	Male	4.08	0.61	16	3.42	1.24	16
		Female	3.45	0.91	14	3.48	0.98	14
		Total	3.79	0.82	30	3.44	1.11	30
	Experimental group	Male	4.10	0.66	7	4.38	0.76	7
		Female	4.39	0.74	6	4.33	0.82	6
		Total	4.23	0.69	13	4.36	0.75	13
	Total	Male	4.09	0.61	23	3.71	1.19	23
		Female	3.73	0.95	20	3.73	1.00	20
		Total	3.92	0.80	43	3.72	1.09	43
Self-concept	Control group	Male	3.61	0.76	16	3.25	1.24	16
		Female	3.16	0.77	14	3.50	0.77	14
		Total	3.40	0.79	30	3.37	1.04	30
	Experimental group	Male	3.75	0.61	7	3.96	0.70	7
		Female	4.21	0.84	6	3.83	1.09	6
		Total	3.96	0.73	13	3.90	0.86	13
	Total	Male	3.65	0.71	23	3.47	1.14	23
		Female	3.48	0.91	20	3.60	0.86	20
		Total	3.57	0.81	43	3.53	1.01	43
Implicit entity theories	Control group	Male	2.52	0.99	16	2.72	1.23	16
		Female	2.79	0.97	14	2.43	0.93	14
		Total	2.64	0.98	30	2.58	1.10	30
	Experimental group	Male	2.14	0.78	7	2.05	0.89	7
		Female	1.50	0.61	6	1.79	1.03	6
		Total	1.85	0.75	13	1.93	0.93	13
	Total	Male	2.40	0.93	23	2.51	1.16	23
		Female	2.40	1.06	20	2.24	0.98	20
		Total	2.40	0.98	43	2.39	1.08	43

In performing the mixed ANOVA to assess the devolvement of interest (H2) in physics between boys and girls in the control and the experimental group, homogeneity of error variances assessed by a Levene test for both measurement points (t1: *p* = 0.25; t2: *p* = 0.16) and homogeneity of covariances, assessed by a Box-test (*p* = 0.63) could be established.

No significant interaction of measurement points and groups, *F*(1, 39) = 0.00, *p* = 0.93, partial *η^2^* < 0.00, or measurement points and genders, *F*(1, 39) = 1.57, *p* = 0.22. *η*_p_^2^ = 0.04, were found.

A statistically significant interaction between measurement points, groups and gender was found, *F*(1, 39) = 6.55, *p* = 0.01, *η*_p_^2^ = 0.14. The partial eta square indicates a medium to large effect. The result suggests that the effect of the intervention differs for male and female students in the control and experimental groups regarding their interest in physics.

No between-subject effects were found for the groups, *F*(1, 39) = 2.40, *p* = 0.13, *η*_p_^2^ = 0.06, gender, *F*(1, 39) = 0.13, *p* = 72, *η*_p_^2^ = 0.0, or group and gender, *F*(1, 39) = 0.01, *p* = 0.91, *η*_p_^2^ < 0.001.

The significant interaction effect was examined through the utilization of profile plots, revealing that students identifying as male exhibited a stronger decline in interest in physics within the control group compared to the experimental group. Conversely, among students identifying as female, an inverse pattern emerged, showcasing a stronger interest decline in the experimental group as opposed to the control group.

When performing the mixed ANOVA to assess the devolvement of self-efficacy (H3) in physics between the control and the experimental group and gender, the Levene test revealed homogeneity of error variances for both measurement points (t1: *p* = 0.65; t2: *p* = 0.26). The Box-test supports the assumption for homogeneity of covariances (*p* = 0.29). No statistically significant interaction between measurement points and groups, *F*(1, 39) = 2,35, *p* = 0.13, *η*_p_^2^ = 0.06, measurement points and gender, *F*(1, 39) = 0.38, *p* = 0.54, partial *η*_p_^2^ = 0.01, or measurement points, groups and gender, *F*(1, 39) = 3.28, *p* = 0.08, *η*_p_^2^ = 0.08, were found.

A significant between-subject effect was found for the groups, *F*(1, 39) = 6.73, *p* = 0.01, *η*_p_^2^ = 0.15, indicating a medium effect size. The result suggests that the intervention groups differ significantly regarding their self-efficacy. No significant between subject effects were found for gender, *F*(1, 39) = 0.09, *p* = 0.76, *η*_p_^2^ = 0.00, or groups and gender, *F*(1, 39) = 0.59, *p* = 0.45, *η*_p_^2^ = 0.02.

The Levene test when conducting the mixed ANOVA for self-concept in physics (H4) revealed homogeneity of error variances for both measurement points (t1: *p* = 0.72; t2: *p* = 0.45). The Box-test supports the assumption for homogeneity of covariances (*p* = 0.14). No significant interaction for measurement points and group, *F*(1, 39) = 0.07, *p* = 0.80, *η*_p_^2^ < 0.001, or measurement points and gender, *F*(1, 39) = 0.04, *p* = 0.84, *η*_p_^2^ < 0.001, were found.

A statistically significant interaction between measurement points, groups and gender was found, *F*(1, 39) = 5.75, *p* = 0.02, *η*_p_^2^ = 0.13. The effect size indicates a medium effect suggesting that the effect of the intervention in regard to the development of self-concepts in physics differs for male and female students in the control and experimental group.

A significant between-subject effect was found for the groups, *F*(1, 39) = 4.47, *p* = 0.04, *η*_p_^2^ = 0.10. No significant between subject effects were found for gender, *F*(1, 39) = 0.02, *p* = 0.90, *η*_p_^2^ < 0.001, or groups and gender, *F*(1, 39) = 0.25, *p* = 0.62, *η*_p_^2^ = 0.01.

The significant interaction effect was further analyzed by profile plots. The visualization revealed a rise in self-concept over time in students identifying as male in the experimental group and a decline for their counterparts in the control group. Conversely, among students identifying as female, an inverse pattern emerged, showcasing a decline in self-concept in the experimental group as opposed to the control group.

Lastly, a mixed ANOVA was conducted to examine students’ implicit entity theories about abilities in physics (H5). The Levene test supported the assumption for homogeneity of error variances for both measurement points (t1: *p* = 0.66; t2: *p* = 0.79). However, the Box-test revealed heterogeneity of covariances (*p* < 0.001), suggesting caution in the interpretation of the results. No statistically significant interaction effects between measurement points and groups, *F* (1, 39) = 0.19, *p* = 0.67, *η*_p_^2^ = 0.01, measurement points and gender, *F*(1, 39) = 0.05, *p* = 0.83, *η*_p_^2^ < 0.001, or measurement points, groups and gender, *F*(1, 39) = 1.35, *p* = 0.25, *η*_p_^2^ = 0.03 were found.

However, there was a significant main effect for groups, meaning that the intervention groups differed significantly in students’ implicit entity theories about abilities in physics, *F*(1, 39) = 8.14, *p* = 0.01, *η*_p_^2^ = 0.17. The partial eta square indicates a large effect.

No significant between-subject effects were found for gender, *F*(1, 39) = 0.78, *p* = 0.38, *η*_p_^2^ = 0.02 or groups and gender, *F*(1, 39) = 0.72, *p* = 0.40, *η*_p_^2^ = 0.02.

## Discussion

The goal of the present study was to test the effects of a game-based VR App in a secondary school physics class during one semester. The study is theoretically based on the actiotope model of motivation (Ziegler et al., 2006, 2011) that explains a person’s goal-oriented actions by an interplay of their environment, action repertoire, and subjective action space. The actiotope model provides a good framework to explain and predict action and engagement in STEM fields which is now needed more than ever before (European Commission, 2022). Enhancing self-beliefs and interest, while supporting academic achievement, are essential and intertwined components of formal education and interventions should be conceptualized in a way to comprise learning holistically. We expected the game-based VR App to have positive effects on students’ action repertoire as measured by their physics performance in three physics tests and their interest in physics compared to traditional teaching methods. Moreover, we anticipated an increase of students’ subjective action space, operationalized as students’ self-efficacy, self-concept, and implicit theories regarding physics, when they used the game-based VR App in addition to traditional learning materials. In sum, we expected that students who used the game-based VR App in addition to traditional learning materials would believe more strongly in their ability to understand and master tasks in physics and show higher interest and better performances in physics. This would be an important prerequisite for students to aspire to careers in physics-related domains. The results only partially confirmed our hypotheses but show some indications of the App effectiveness.

Results regarding performance in our study (H1) showed no significant differences between the experimental and control groups on tests related to the first two thematic units (“noble and base metals” and “world of light”), but the experimental group achieved significantly better results, compared to the control group, on the third test (related to the thematic unit “radioactivity”).

Regarding the development of students’ interest in physics (H2), we did not find differences between the experimental group and the control group. This indicates that in general, students using the game-based VR App did not show a stronger increase or lower increase in interest than students in the control group who did not use the App. However, we found differential effects of the intervention on interest development in students of different genders as indicated by a three-way interaction of gender, experimental condition, and time. In students identifying as male, interest in physics declined over the semester in the control group but not in the experimental group, which aligns with our hypothesis that the game-based VR App has more positive effects on students’ action repertoire than traditional teaching methods. However, in students identifying as female, we found the opposite pattern. Although the effects are rather small, this could indicate that game-based virtual reality teaching methods favor students identifying as male. This might be explained by boys’ higher engagement with video games in general (Drabowicz, 2014) and especially with 3D-animated computer games. Namely, studies show that female players are more attracted to 2D games while male players are more attracted to 3D games (e.g., Ziemek, 2006).

To assess the development of students’ subjective action space, we analyzed changes in students’ self-efficacy, self-concept, and entity implicit theories regarding physics over the course of the semester. While domain-specific self-efficacy and self-concept are facets of students’ physics-related self-image, entity implicit theories refer to the extent to which they generally believe that abilities in physics are unchangeable. Contrary to our hypothesis (H3), we found no significant differences in the development of students’ self-efficacy over the course of the semester between the control group and the experimental group. However, the effect sizes indicate medium differential effects of the intervention in students of different genders, as indicated by a marginally significant (<0.1) three-way interaction of gender, experimental condition, and time. Male students’ self-efficacy in physics tends to decline over the semester in the control group but not in the experimental group, while in female students’ self-efficacy did not develop differently in the two groups. However, this finding – although in line with our finding regarding students’ interest in physics – should be interpreted cautiously.

Regarding students’ domain-specific self-concept in physics (H4), we also did not find what we expected. Contrary to our expectations, students using the game-based VR App did not show a more positive development of their physics self-concept than students in the control group who did not use the App. However, we again found differential effects of the intervention in students of different genders as indicated by a three-way interaction of gender, experimental condition, and time. Boys’ self-concept in physics declined over the semester in the control group but not in the experimental group, which aligns with our hypothesis that the game-based VR App has more positive effects on students’ subjective action space than traditional teaching methods. However, in students identifying as female, we again found the opposite pattern. Contradicting our last hypothesis, no significant effects of the intervention were found regarding the development students’ implicit entity theories (H5).

Taken together, our findings indicate that the App might have a slight positive effect, and more so on male than female students’ motivation in physics. This result only partly aligns with a large body of literature showing unambiguous positive effects of game-based VR on psychological learning outcomes in physics (e.g., Pellas et al., 2020; Pirker et al., 2020). However, it is important to note that the App was not used as much as we hoped as can be seen in the user data analysis. The pandemic might be a reason for the generally low participation rate in the App use. As it has been found that students struggled to adequately organize and self-regulate their learning during the pandemic (Pelikan et al., 2021; Holzer et al., 2021), they might have been overwhelmed with learning processes and challenges concerning health behavior which limited their resources to use the App more. Nevertheless, our study suggests that incorporating game-based VR learning materials into secondary school physics class could potentially exacerbate gender gaps in physics in these early stages of students’ acquainting with physics. This could be since both physics as a school subject (Kessels et al., 2006) and the innovative learning material of a VR App are related to masculine stereotypes (Ziemek, 2006). Research shows that gender disparities in STEM emerge in this period of adolescence when students are about to move into high school and choose their education and potentially career track (e.g., Osborne, 2007). Consequently, young girls are still less likely than boys and young men to engage in courses and majors related to physics and science (Moss-Racusin et al., 2018). Thus, when developing and implementing innovative teaching materials, findings like ours should be considered.

## Limitation and future directions

Several limitations of the present study should be noted. In general, the sample of our pilot study, especially in the control group, is very small, and the App was not used very much by students in the experimental group. Therefore, our results must be interpreted with caution and can only be seen as initial indications of the effectiveness of the intervention.

Further research with larger samples can learn from some methodological shortcomings of our study: First, since the App was developed only for one operating system, students could not be fully at random assigned to the experimental and control group limiting the generalizability of our results. Students with iPhones and those without phones assigned to the control group might differ from students with Android phones in terms of their socioeconomic status. Therefore, future studies should aim at developing applications for all common operating systems. Second, only a portion of students assigned to the experimental group used the App, but the context of the data collection in the middle of the COVID-19 pandemic as well as decreased control in the natural experimental environment prevented any tries to increase the participation rate. Third, our assessment of students’ physics performance could have been optimized if we assessed domain-specific prior knowledge tests before the intervention and conducted a more objective performance test with different items after the intervention. In relying on the tests designed by the teacher, we maybe did not assess students’ physics competencies, but rather how well they learned the test answers. Unfortunately, when cooperating with schools, we are restricted in how much data we can collect. Every data collection must be squeezed in by the teachers who are afraid to lose precious time for fulfilling the curriculum. This problem was exacerbated during the early COVID-19 pandemic (with the rather confusing situation of school closures, reduced group sizes and changing attendance of students). Fourth, we did not assess all actiotope variables with standardized scales. Although the internal consistencies of our shortened and/or adapted scales were good, future studies should not only assess students’ action repertoire and subjective action space but also their goals to be able to make better statements about the effects of the intervention on educational and career goals. Finally, future research should also include possible covariates such as student’s previous achievement level (e.g., Keller et al., 2021), attitudes toward, experiences with, and access to digital media, or teachers’ knowledge and motivation (Keller et al., 2017).

## Conclusion and practical implications

Albeit its limitations, this quasi-experimental study contributes to the body of literature showing that providing students with opportunities to experience physics phenomena in game-based VR environment can potentially have positive effects performance of students in secondary school physics class. Schools should provide students with a broader set of 21st century skills, which are labeled as survival skills in new generations (Saavedra and Opfer, 2012), to thrive in a rapidly evolving, technology-saturated world. The simple technical solutions, such as mobile VR with Google cardboards can be considered as low cost (Pellas et al., 2020) and might be available to most digital native learners as mobile applications, smartphones, and digital environments are a part of their daily lives (Hussein and Natterdal, 2022). However, using these technologies as teaching materials might potentially increase gender differences in self-beliefs in a period crucial for development of career and educational aspirations and choices if no explicit attention is paid to this problem. Moreover, equal opportunities for students with different socio-economic and migration backgrounds should be ensured. Researchers should focus on multiple learning outcomes, including psychological variables (such as self-beliefs, interest and motivational factors, etc.), to investigate how educational games and VR contribute to learning broadly. Moreover, interdisciplinary approaches (e.g., cooperation between psychologists, physicists and computer scientists) should be practiced in order to develop successful and innovative interventions in this field.

## Data availability statement

The raw data supporting the conclusions of this article will be made available by the authors, without undue reservation.

## Ethics statement

Ethical approval was not required for the studies involving humans because the local legislation and institutional requirements do not require one. Participation in the study was completely voluntarily. Participants were informed about the approximate duration of the questionnaires/tests, inclusion criteria for participation, and the complete anonymity of their data. Only those who gave active consent were included in the dataset. The study was carried out in accordance with the European General Data Protection Regulation. The studies were conducted in accordance with the local legislation and institutional requirements. Written informed consent for participation was not required from the participants or the participants’ legal guardians/next of kin in accordance with the national legislation and institutional requirements because data was collected online due to circumstances of the COVID-19 pandemic and only consent from the students themselves was collected. Written informed consent was obtained from the individual(s) for the publication of any potentially identifiable images or data included in this article.

## Author contributions

SK: Conceptualization, Data curation, Formal analysis, Investigation, Methodology, Project administration, Validation, Visualization, Writing – original draft, Writing – review & editing. MK: Visualization, Writing – review & editing, Conceptualization. CH: Writing – review & editing, Formal analysis. HH: Conceptualization, Investigation, Writing – review & editing, Funding acquisition, Project administration, Resources, Supervision. DM: Conceptualization, Investigation, Software, Writing – review & editing. PP: Conceptualization, Investigation, Writing – review & editing, Software. CS: Conceptualization, Funding acquisition, Investigation, Project administration, Resources, Writing – review & editing.
