# Supplementary material for: PhyLab – a virtual reality laboratory for experiments in physics: a pilot study on intervention effectiveness and gender differences
Source: Front Psychol. 2024 Feb 19;15:1284597. doi: 10.3389/fpsyg.2024.1284597 (PMC10909838; doi:10.3389/fpsyg.2024.1284597)
Supplement: Supplementary file 1 [file Data_Sheet_1.docx]

**Online Supplementary Materials**

Figure S1. Experiment on electricity - selection of different materials to make a battery. GerthMichael, CC BY-SA 3.0 <[http://creativecommons.org/licenses/by-sa/3.0/>](http://creativecommons.org/licenses/by-sa/3.0/%3e), via [Wikimedia Commons](https://commons.wikimedia.org/wiki/File:Sgdn_1.jpg).
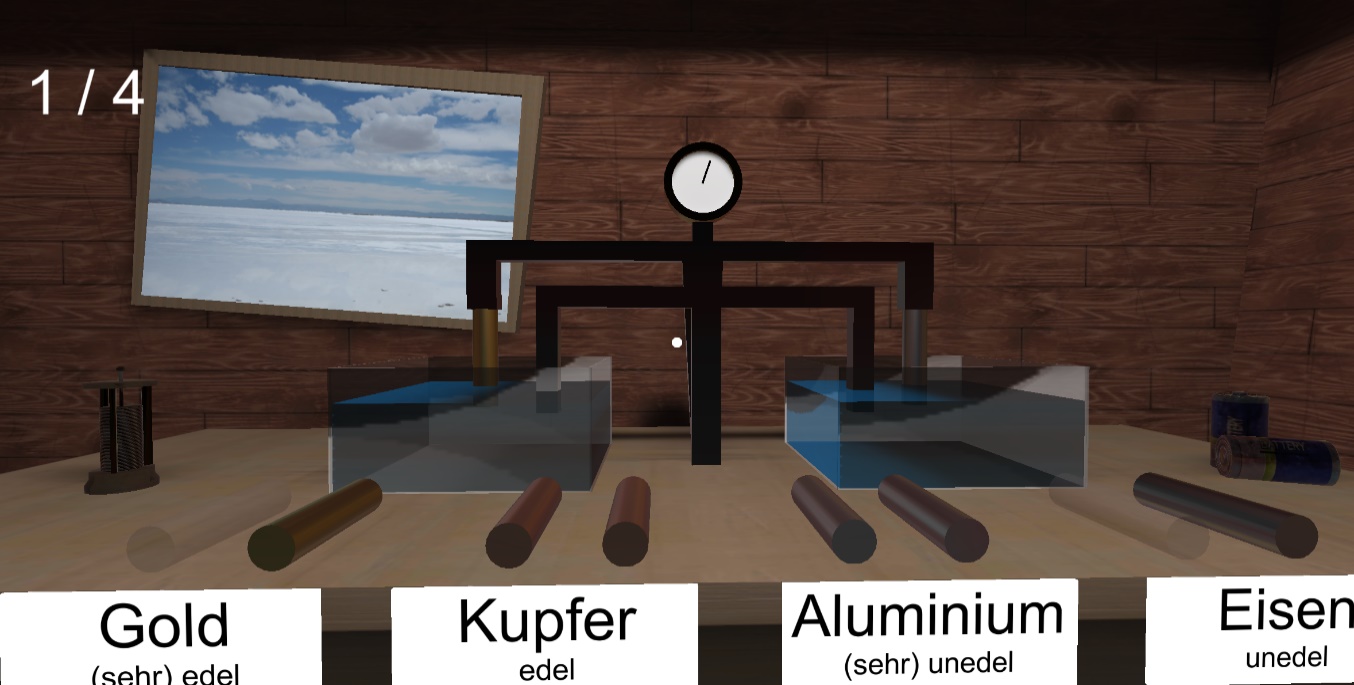


Figure S2. Further information about Alessandro Volta and his work in the field of electricity
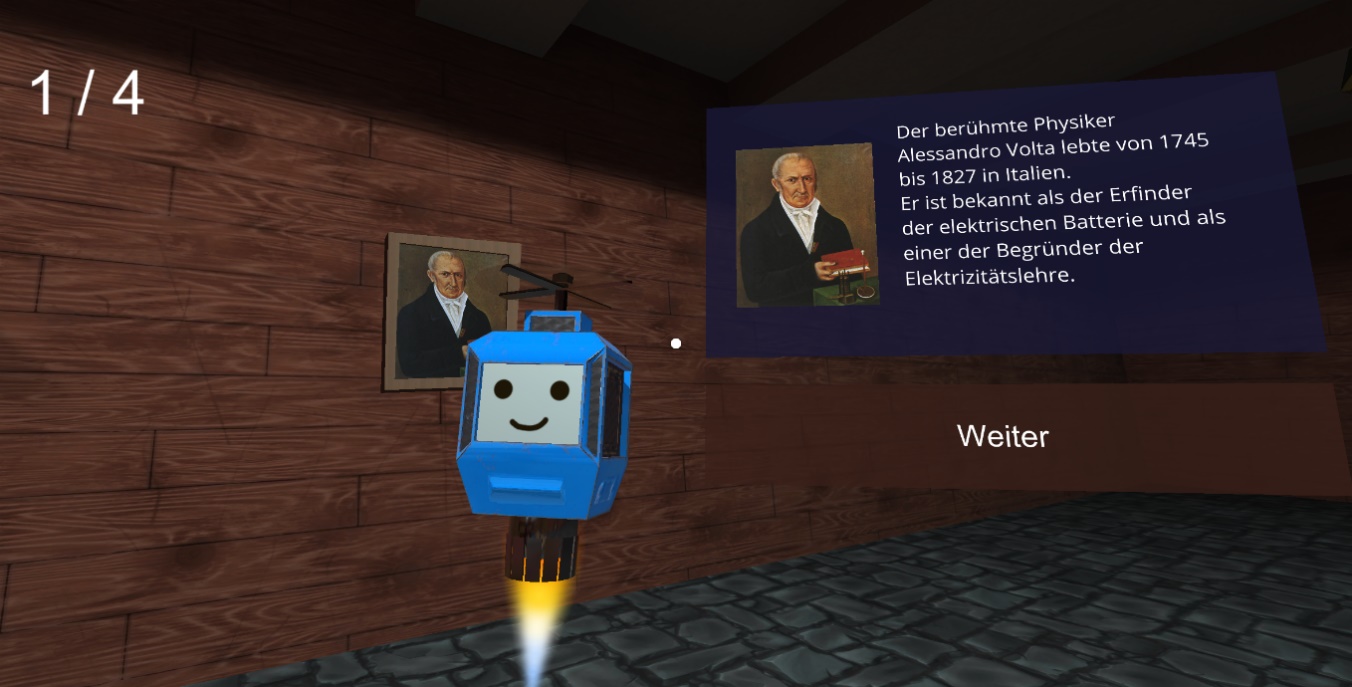


Figure S3. Further information about the voltaic column in the field of electricity.

Luigi Chiesa, CC BY-SA 3.0 <[https://creativecommons.org/licenses/by-sa/3.0>](https://creativecommons.org/licenses/by-sa/3.0%3e), via [Wikimedia Commons](https://commons.wikimedia.org/wiki/File:Pila_di_Volta_01.jpg). **
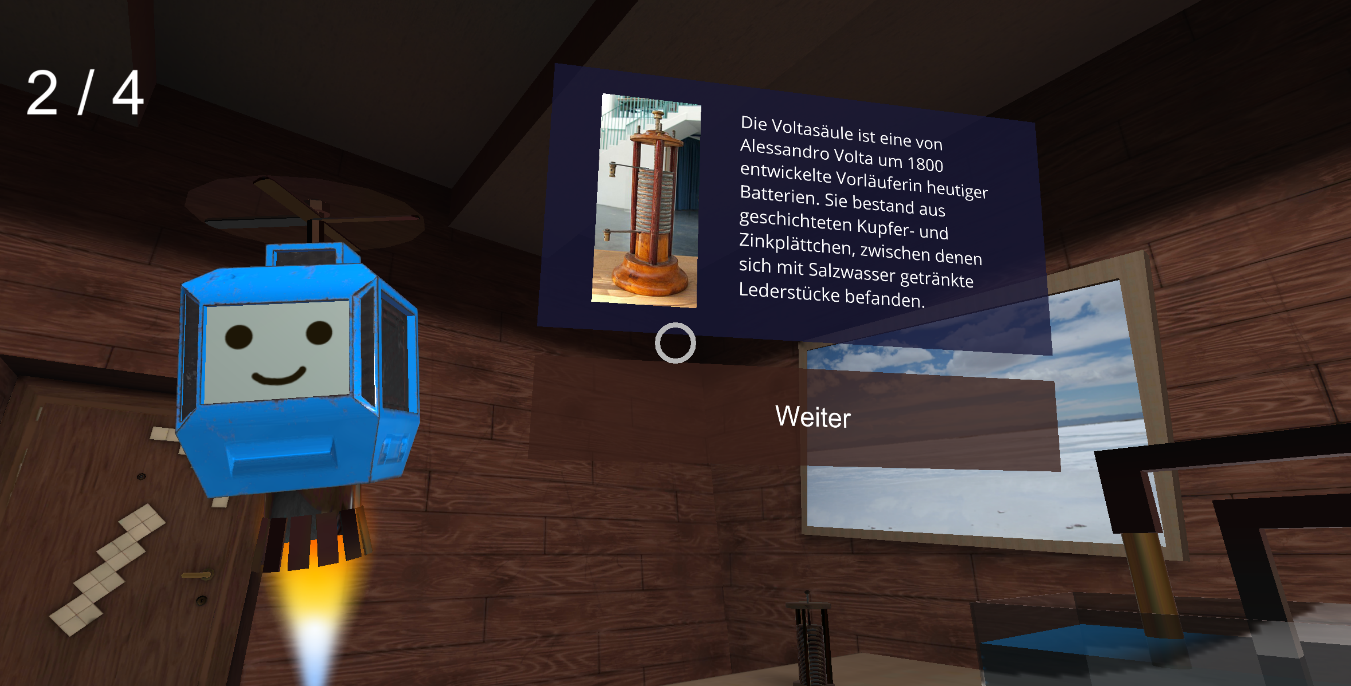
**

Figure S4. Experiment on the electromagnetic spectrum - selection of one of the different light spectra (the laptop on the left emits infrared radiation)


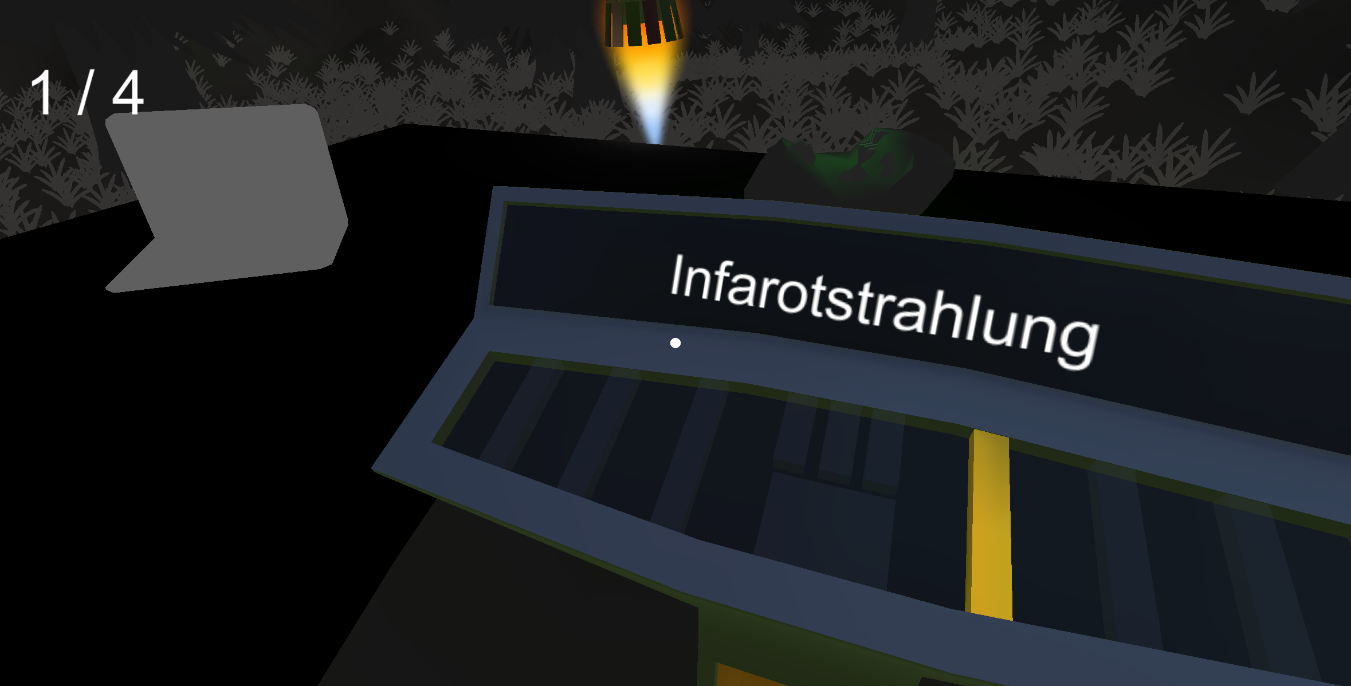


Figure S5. Experiment on the electromagnetic spectrum - select gamma radiation and show that the uranium ore emits the radiation
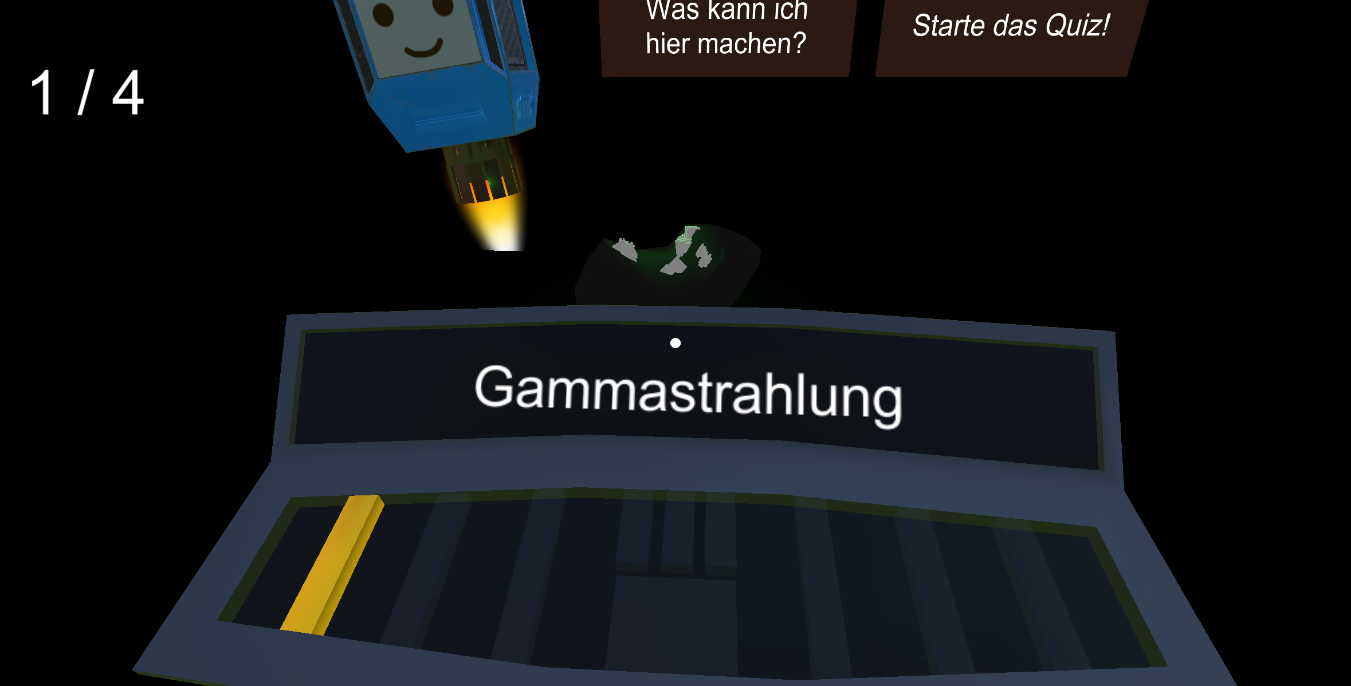


Figure S6. Further information on radiation exposure in the field of the electromagnetic spectrum**
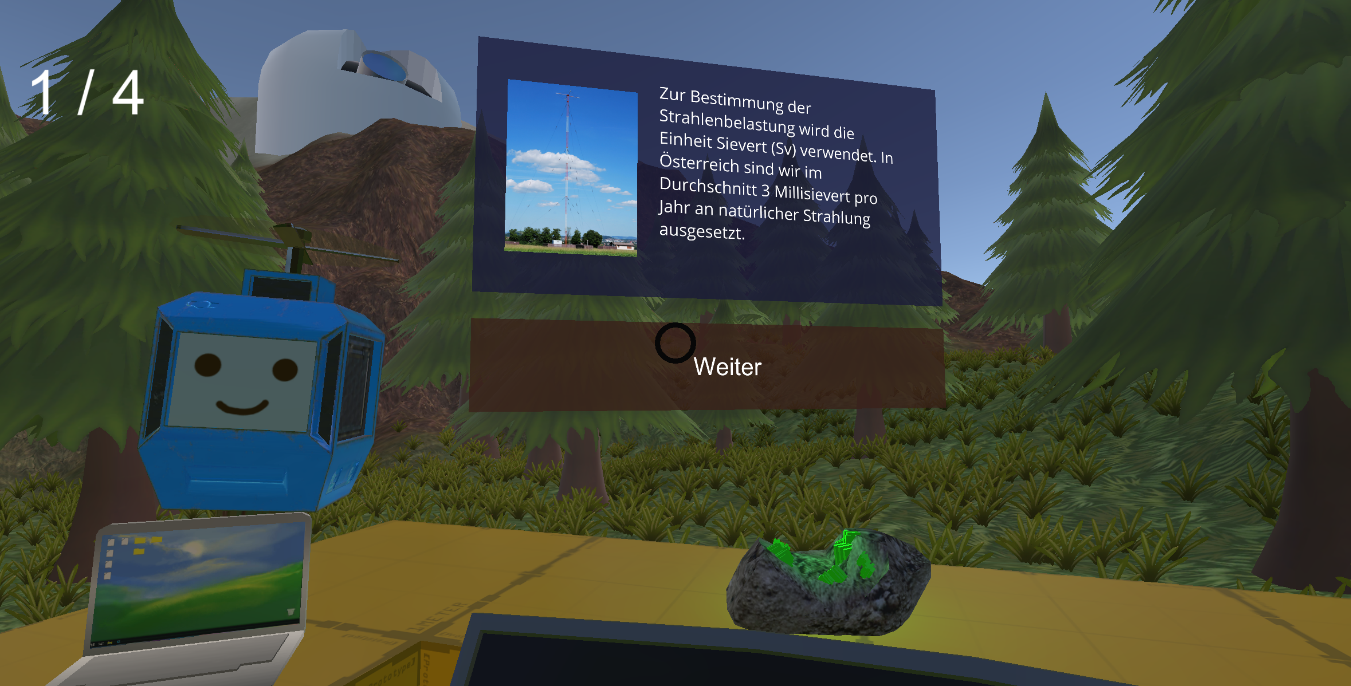
**

Figure S7. Experiment on radiation - inserting an aluminum plate to stop alpha radiation**
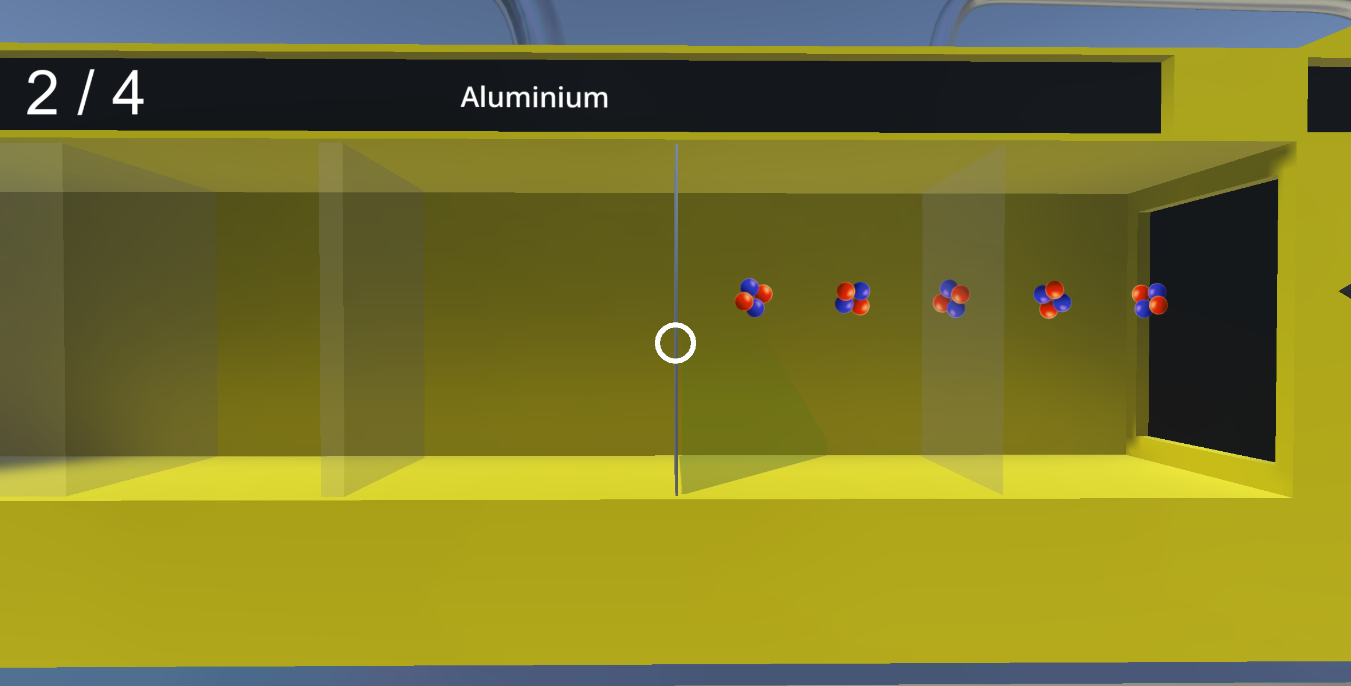
**

Figure S8. Experiment on radiation - gamma radiation penetrates the aluminum barrier
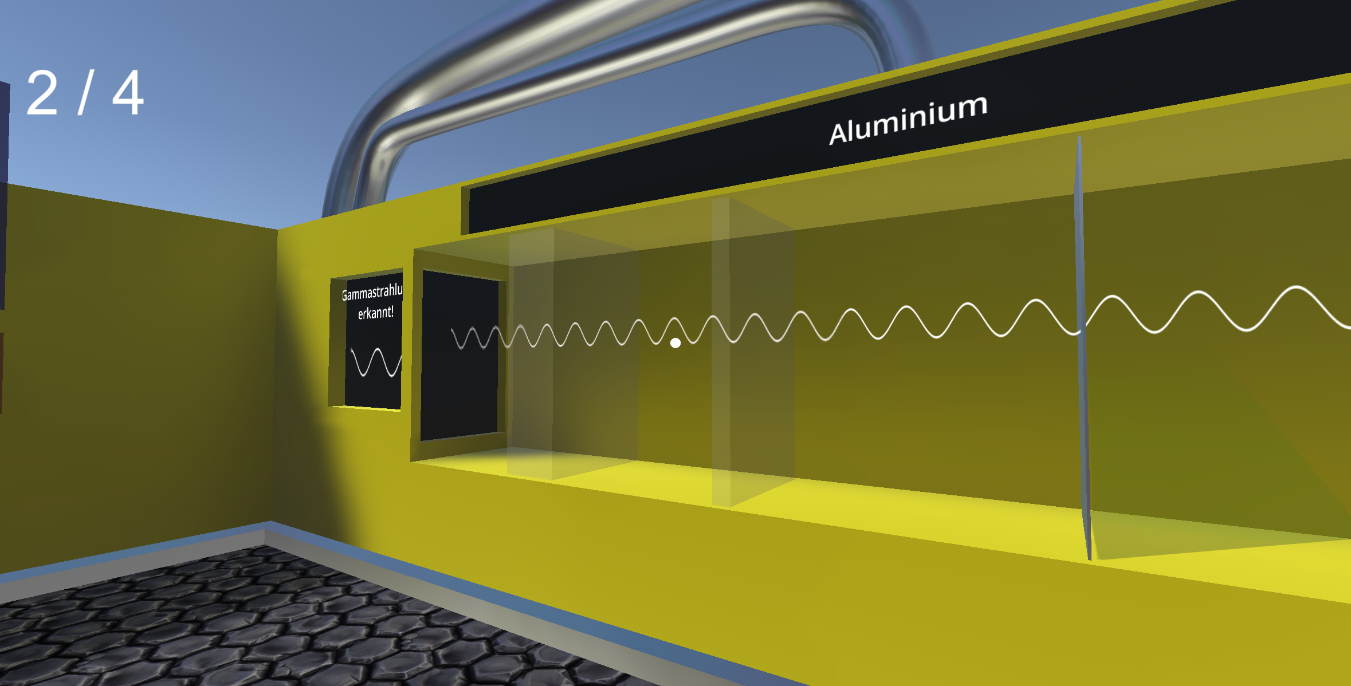


Figure S9. Further information on Lise Meitner and her work in the field of radioactivity.

Kvikk, CC BY-SA 4.0 <[https://creativecommons.org/licenses/by-sa/4.0>](https://creativecommons.org/licenses/by-sa/4.0%3e), via [Wikimedia Commons](https://commons.wikimedia.org/wiki/File:Lise_Meitner_Denkmal_Unter_den_Linden_Berlin_(3).JPG).


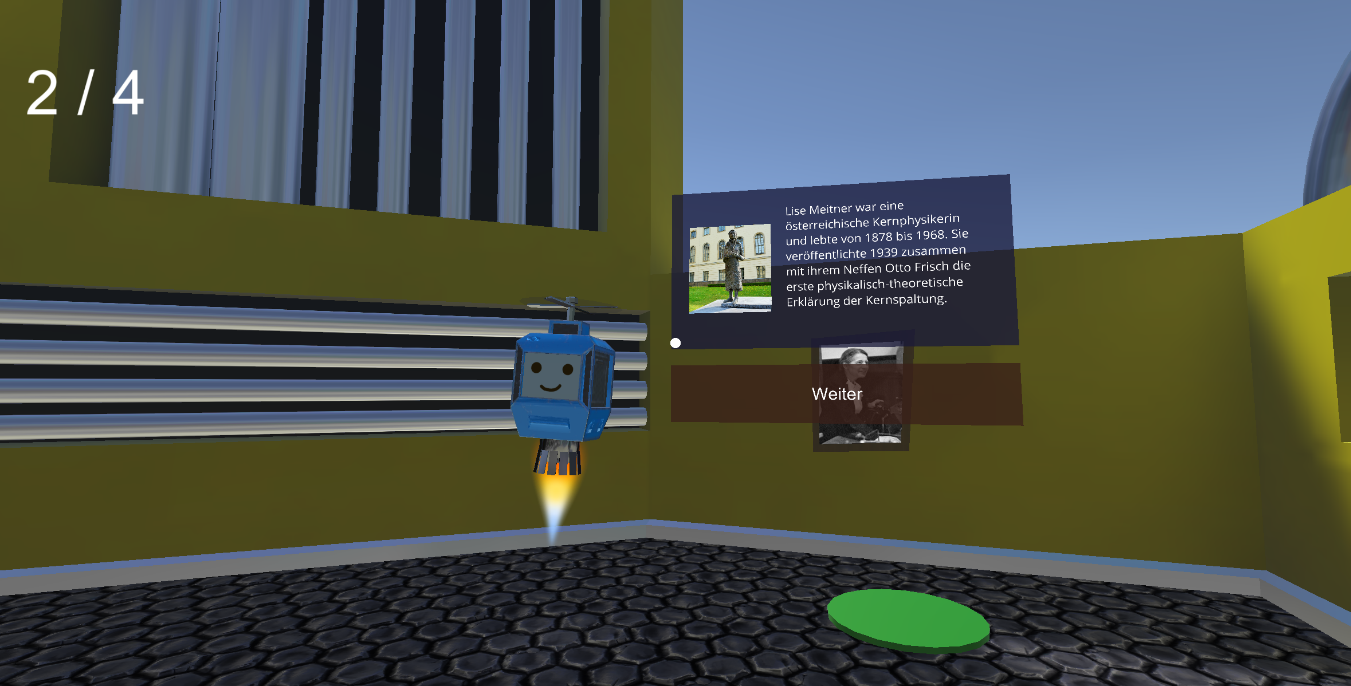


T1. Sample task for test 1 (related to the teaching unit "Precious and base metals"*)

Various metals in a weak hydrochloric acid solution (HCl) generate an electrical voltage. The metal that "generates" the positive pole is the more noble metal.

Assign metals based on their poles and insert their voltage:

Measurement values:

| Positive Pole + | Negative Pole - | Voltage (U) in Volt (V) |
| --- | --- | --- |
|  |  |  |
|  |  |  |
|  |  |  |
|  |  |  |
|  |  |  |

The greater the electrical voltage between the metals, the greater the difference between the precious and base metal. Insert metals based on this:

Order of metals

| 1. |  |  | 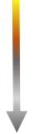 | precious |
| --- | --- | --- | --- | --- |
| 2. |  |  |  |  |
| 3. |  |  |  |  |
| 4. |  |  |  |  |
| 5. |  |  |  | base |

T2. Sample task for test 2 (related to the teaching unit "World of light")

**The colors of our environment are created by the colors of the light source that illuminates the environment and by different materials that reflect or absorb ("swallow") certain colors.**

**An overview of the perception of colors under different light conditions:**

| **Colours of objects:** | **Color of the LED light** | | | | | | |
| --- | --- | --- | --- | --- | --- | --- | --- |
|  | **no light** | **cold-white** | **warm-white** | **red** | **green** | **blue** | **ultraviolet** |
| **black** |  |  |  |  |  |  |  |
| **white** |  |  |  |  |  |  |  |
| **red** |  |  |  |  |  |  |  |
| **orange** |  |  |  |  |  |  |  |
| **yellow** |  |  |  |  |  |  |  |
| **green** |  |  |  |  |  |  |  |
| **blue** |  |  |  |  |  |  |  |
| **fluorescent colour** |  |  |  |  |  |  |  |
| **Tonic-Water** |  |  |  |  |  |  |  |

T3. Sample task for test 3 (related to the teaching unit "Radioactivity")

**Different substances can stop (absorb, filter) radioactivity:**

If the type of radiation passes through the substance, write "P" for passage.

If the type of radiation does not pass through the substance, write "A" for absorption.

| **Type of radiation** | **Substance** | | | |
| --- | --- | --- | --- | --- |
|  | **water** | **lead** | **aluminium** | **paper** |
| **α-radiation** |  |  |  |  |
| **β- radiation (elektrons)** |  |  |  |  |
| **γ- radiation** |  |  |  |  |
| **neutrons** |  |  |  |  |
